# Primary PreserFlo MicroShunt Versus Trabeculectomy: Effectiveness and Safety in the Real World

**DOI:** 10.3390/jcm14217484

**Published:** 2025-10-22

**Authors:** Anoushka N. Kothari, Graham A. Lee

**Affiliations:** 1 Mayne Academy of Surgery, University of Queensland, Brisbane, QLD 4006, Australia; 2 Brisbane North Eye Centre, Chermside, QLD 4032, Australia

**Keywords:** glaucoma, trabeculectomy, PreserFlo MicroShunt, intraocular pressure, surgical complications

## Abstract

**Backgrounds/Objectives:** Trabeculectomy is the gold standard for glaucoma drainage surgery, but it is associated with a risk of sight-threatening complications. The PreserFlo MicroShunt (PF) is a less invasive alternative that aims to reduce complications and simplify post-operative care. This study aimed to compare the effectiveness and safety of PF to trabeculectomy in the management of glaucoma. **Methods:** This was a retrospective cohort analysis of 95 eyes (48 PF, 47 trabeculectomy) from a single-center private practice in Brisbane, Australia. Data were collected from November 2017 to January 2024. Primary outcomes included intraocular pressure (IOP) and the number of medications. Secondary outcomes included best-corrected visual acuity (BCVA) and complications. Inverse probability of treatment weighting (IPTW) was applied to baseline covariates, and weighted regression and Cox proportional hazards models were then used to estimate treatment effects. **Results:** The two groups had comparable patient characteristics, although the PF group was older with worse visual field mean deviation. At 12 months, both procedures significantly reduced IOP and medications; however, differences were not statistically significant between groups (2.9 mmHg; 95%CI: −2.0, 7.9; *p* = 0.303, and 0.4; 95%CI: −0.13, 0.96; *p* = 0.138, respectively). The estimated probabilities of qualified success were comparable (74.9% PF vs. 72.5% trabeculectomy). Intra-operative stenting in PF eyes eliminated early post-operative hypotony. The incidence of open surgical revision in the PF group vs. the trabeculectomy group was 14.6% vs. 2.1% (*p* = 0.059, respectively). PF was associated with faster post-operative inflammation resolution (hazard ratio: 6.3; 95%CI: 2.8, 14.5; *p* < 0.001). **Conclusions:** Both PF and trabeculectomy are effective for glaucoma management. PF is a less invasive procedure with a lower rate of early hypotony when stented. Trabeculectomy has a tendency for lower IOP reduction and less requirement for open revision, although this did not reach statistical significance. This highlights the need for longer-term studies and improved techniques, such as more effective anti-fibrotic strategies.

## 1. Introduction

Trabeculectomy is considered the gold standard for drainage surgery in glaucoma. It is an effective procedure for lowering intraocular pressure (IOP) and reducing the need for post-operative medication. However, due to its invasive nature, trabeculectomy surgery carries a risk of sight-threatening post-operative complications. These complications prolong recovery and may even require surgical revision. Surgeons also require a high level of skill and expertise to perform trabeculectomy surgery [1,2,3,4,5].

There has been a shift towards less invasive surgical procedures, such as the PreserFlo MicroShunt (PF) (Santen, Osaka, Japan). This device aims to avoid the need to create a scleral flap, sclerostomy, and pre-placed nylon sutures, as well as to reduce post-operative complications and follow-up review requirements. The reduced intra-operative trauma results in less post-operative inflammation and potentially better preservation of tissues for future surgery [1,2,3,4,5].

This study aimed to compare the effectiveness and safety of PF to trabeculectomy in the management of glaucoma.

## 2. Materials and Methods

This is a retrospective cohort analysis of consecutive patients undergoing glaucoma drainage surgery at a single-center private ophthalmology practice by a single surgeon (G.A.L.) in Brisbane, Australia. Surgeries were conducted between November 2017 and October 2022 for trabeculectomy patients and between May 2022 and January 2024 for PF patients. Written consent for surgery was obtained from all patients with institutional ethical approval (Royal Australian and New Zealand College of Ophthalmology 195.25). The inclusion criteria involved patients aged ≥18 years with mild to advanced glaucoma, which was uncontrolled on maximally tolerated medication.

Data was retrospectively collected pre-operatively as well as post-operatively on day 1, week 1, and at 3, 6, and 12 months. Information on the patient’s age, sex, ethnicity, type of glaucoma, and past ocular history was recorded. Primary outcome measures included IOP and the number of IOP-lowering medications. Secondary outcome measures included best-corrected visual acuity (BCVA), visual field mean deviation (MD), retinal nerve fiber layer thickness, and intraluminal stent removal (for PF patients). The severity of glaucoma was based on the Hodapp–Parrish–Anderson classification of MD with mild ≥ −6 dB, moderate between −6–−12 dB and severe ≤ −12 dB. Data was also collected on any post-operative complications, including the need for revision surgery.

The criteria for complete success were an IOP between 6 mmHg and 15 mmHg, with a ≥20% reduction in IOP from baseline, no requirement for post-operative IOP-lowering medication, and no subsequent procedures, revisions, or additional glaucoma surgeries. Qualified success was defined as an IOP between 6 mmHg and 15 mmHg, with a ≥20% reduction in IOP from baseline, regardless of the use of adjunct medication (but not including acetazolamide), and no further glaucoma procedures or surgeries. Any additional glaucoma surgery, except for needling with 5-fluoruracil, during the follow-up period was defined as a failure.

**Figure 1:**
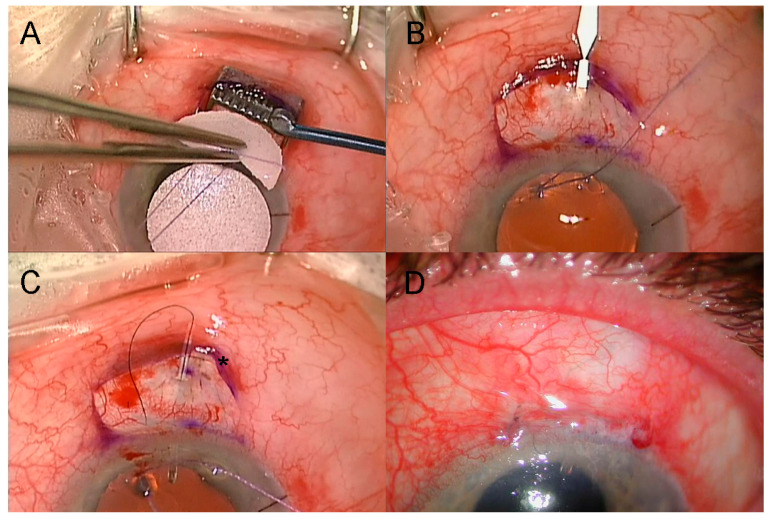
Routine insertion of PreserFlo Microshunt. (**A**) Application of mitomycin-C with half-circle PVA sponges using conjunctival retractor. (**B**) Two-step knife to create sclerostomy, commencing 4mm behind limbus. (**C**) 10/0 nylon intraluminal stent with releasable loop buried in peripheral corneal tunnel (*). (**D**) Closure of limbal wound with buried interrupted 9/0 polyglactin horizontal mattress sutures.

For the PF surgery, the procedure was performed with a fornix-based conjunctival flap, followed by the application of Mitomycin C (MMC) at 0.01–0.04% for 2–3 min (Figure 1A), depending on the state of the conjunctiva and Tenon’s capsule as determined by the surgeon (G.A.L.). The device was placed ab externo with the formation of the sclerostomy using a two-step knife (Mani, Tochigi, Japan), commencing 4 mm behind the scleral limbus (Figure 1B). A buried releasable 10/0 nylon intraluminal stent was placed after the first eight eyes that had PF insertion (Figure 1C). The conjunctiva was closed with four buried 9/0 polyglactin 910 (Vicryl, Ethicon, Inc., Raritan, NJ, USA) buried horizontal mattress sutures (Figure 1D). Subconjunctival dexamethasone and cefazolin were injected.

For the trabeculectomy surgery, a fornix-based conjunctival flap was created, followed by a scleral flap (4 mm × 3 mm × 300 microns thick), and then the application of MMC at 0.01–0.04% for 2–3 min, depending on the state of the conjunctiva and Tenon’s capsule as determined by the surgeon (G.A.L.). A peripheral iridectomy was performed in all phakic patients and in pseudophakic patients if the iris base was occluding the sclerostomy. The flap was closed with two or three triple-throw buried releasable 10/0 nylon sutures, and the conjunctiva was closed with a combination of two purse-string and 3–4 10/0 nylon buried horizontal mattress sutures. A Seidel test was performed to confirm no leaks. Subconjunctival dexamethasone and cefazolin were injected.

Post-operatively, patients were commenced on topical prednisolone acetate 1% and antibiotic eye drops six times per day. The topical antibiotic was ceased after 1–2 weeks, and the topical steroids were gradually tapered over 4–6 months. IOP-lowering medication was not administered unless indicated.

Statistical analyses were conducted using Stata version 18 (StataCorp LLC, College Station, TX, USA). Differences in means between groups were analyzed with an independent t-test. Between-group differences in proportions and differences in categorical distributions between treatment groups were analyzed using Fisher’s exact test or the Chi-squared test. To account for the inherent correlation between repeated measurements within individual eyes and the inclusion of both eyes from the same patient, multilevel mixed-effects linear or Poisson regression models were employed, controlling for age, pre-operative IOP, number of glaucoma medications, and MD. Inverse probability of treatment weighting (IPTW) was applied using propensity scores estimated from the same covariates included in the regression models to mitigate potential selection bias between groups. The hazards of failure to achieve success criteria and of ceasing steroid use were analyzed using IPTW-Cox proportional hazard modelling, controlling for age, pre-operative IOP, the number of glaucoma medications, and MD. *p* < 0.05 indicated statistical significance.

## 3. Results

A total of 95 eyes (48 PF and 47 trabeculectomy) in 83 patients (44 PF and 39 trabeculectomy) were included in the study. The groups had comparable characteristics with a few exceptions (Table 1). The PF group was older, with a mean ± standard deviation age of 72.9 ± 9.6 years compared to 65.3 ± 13.8 years in the trabeculectomy group (*p* = 0.002). The MD was worse in the PF group at −11.1 ± 7.8 dB compared to −7.3 ± 6.6 dB in the trabeculectomy group (*p* = 0.014). The initial eight PF procedures were performed without the placement of an intra-operative intraluminal 10/0 nylon stent. In the subsequent 40 cases, stents were placed, with 31 (77.5%) of these removed within three months post-operatively. The PF group received slightly higher doses of MMC, with 29 (60.4%) receiving a concentration of 0.03% for 3 min, compared to 20 (42.6%) in the trabeculectomy group (*p* = 0.131).

Outcome analysis was conducted using the IPWA results to balance out differences between the groups without reducing the sample size. Standardized mean differences were close to zero, with a range of weighted standard differences between −0.04 and 0.01, indicating excellent covariate balance, and variance ratios were within the acceptable range of between 0.6 and 1.4.

**Figure 2:**
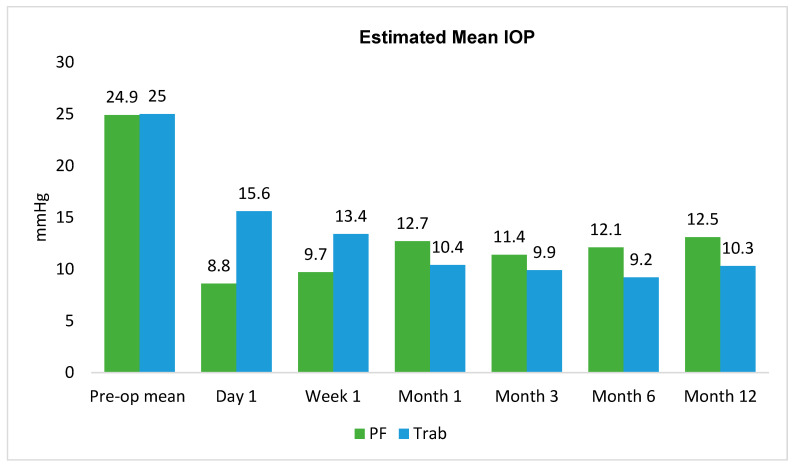
Estimated mean IOP for the 12-month follow-up period using inverse probability weighted adjustment.

The mean IPWA-adjusted pre-operative IOP was 24.9 mmHg in the PF group and 25.0 mmHg in the trabeculectomy group (Table 2, Figure 2). The difference in mean IOP reduction from baseline was statistically significantly greater in the PF group at day 1 and week 1, with no significant differences between groups at subsequent time points. At Month 12, the mean IOP was 13.1 mmHg in the PF group, resulting in an IOP reduction of −11.8 mmHg from baseline. In the trabeculectomy group, the mean IOP was 10.3 mmHg, with a reduction of −14.7 mmHg. The between-group difference in IOP reduction was 2.9 mmHg (95%CI: −2.0, 7.9; *p* = 0.246).

**Figure 3:**
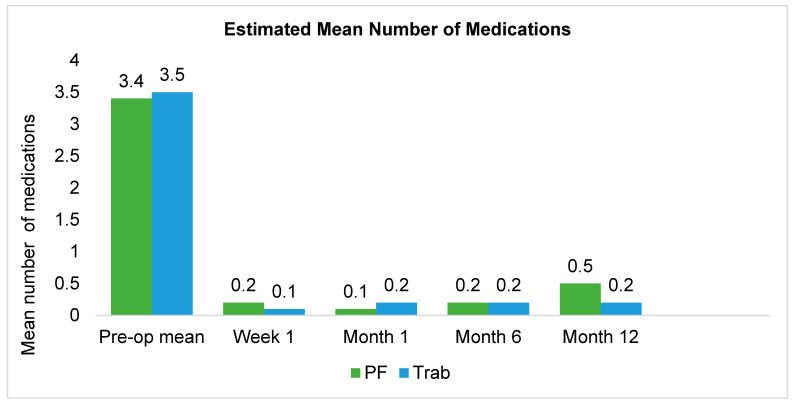
Estimated mean number of medications for the 12-month follow-up period using inverse probability weighted adjustment.

Over the 12-month study period, on average, the PF group exhibited a reduction of 2.9 medications, whilst the trabeculectomy group had a reduction of 3.3 medications, resulting in a between-group difference in medication reduction of 0.4 (95%CI: −0.13, 0.96; *p* = 0.138). At 12 months, the mean number of post-operative medications for the PF group was 0.5 and 0.2 medications in the trabeculectomy group (Figure 3, Table 3).

The IPWA-adjusted mean BCVA in the PF and trabeculectomy eyes pre-operatively were both 0.76, and by 12 months post-operatively, the PF eyes had the same mean BCVA at 0.76, whereas the trabeculectomy group was 0.73 (between-group differences: 0.03; 95%CI: −0.1, 0.2; *p* = 0.710).

Six of the eight (75.0%) non-stented PF eyes developed early (≤1 month) post-operative numerical hypotony. This prompted the subsequent 40 eyes to have intra-operative placement of a 10/0 nylon intraluminal stent, with no further episodes of hypotony. Among the non-stented eyes, one eye (12.5%) experienced clinically significant hypotony requiring re-stenting at one month. Further complications are detailed in Table 4. In the trabeculectomy eyes (*n* = 47), early post-operative complications included numerical hypotony in six eyes (12.8%), macroscopic hyphema in one eye (2.1%), peripheral iris incarceration in one eye (2.1%), and bleb leak in two eyes (4.7%).

Bleb needling with 5% 5-fluorouracil was required in both treatment groups, with 16 (33.3%) PF eyes and 13 (27.7%) trabeculectomy eyes. Needling was performed at a mean of 2.6 months post-operatively in the PF eyes and 1.9 months post-operatively in trabeculectomy eyes, suggesting a requirement for earlier post-operative bleb management in the trabeculectomy group (Table 4).

The cessation of topical steroids (based on clinical observation of bleb appearance, such as vascularity, extent, and height) in PF was 6.3 times (95%CI: 2.8, 14.5; *p* < 0.001) more likely than in trabeculectomy, indicating faster resolution of post-operative inflammation following PF surgery. The estimated cumulative probability of continued steroid use at Months 6 and 12 was 48.3% and 10.5% in the PF group, compared with 89.2% and 70.1% in the trabeculectomy group, respectively.

**Figure 4:**
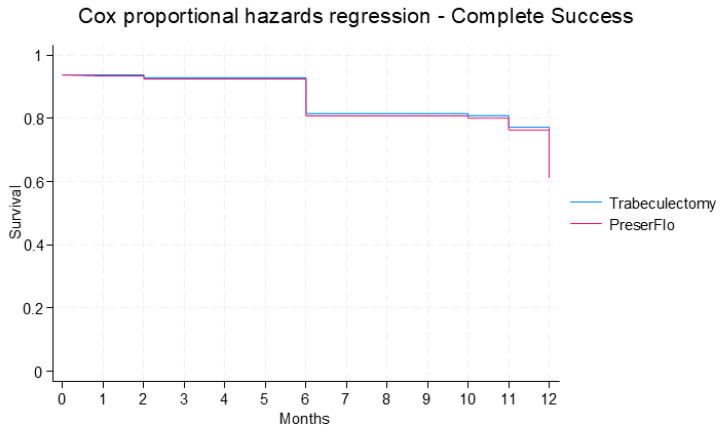
Cumulative probability of complete success in PF and trabeculectomy groups estimated from Cox proportional hazard model.

**Figure 5:**
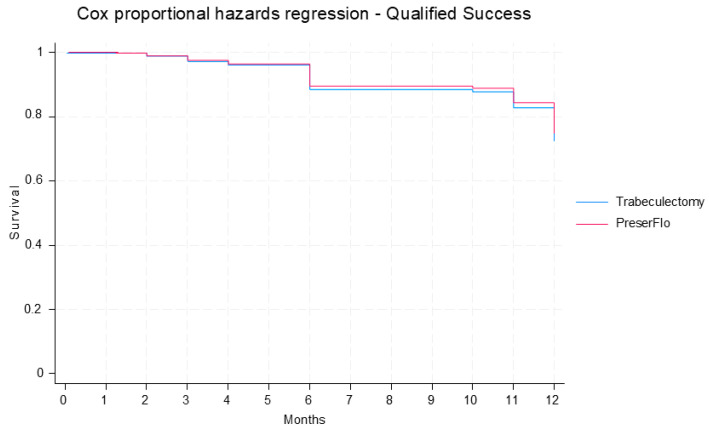
Cumulative probability of qualified success in PF and trabeculectomy groups estimated from Cox proportional hazard model.

Complete and qualified success did not differ significantly between groups. The cumulative probability of complete success was estimated to be 61.3% in the PF group and 62.7% in the trabeculectomy group at 12 months post-operatively [hazard ratio (HR): 1.0; 95% CI: 0.5, 2.2; *p* = 0.984) (Figure 4). The cumulative probability of qualified success was predicted to be 74.9% in the PF group and 72.5% in the trabeculectomy group at 12 months post-operatively (HR: 0.9; 95% CI: 0.4, 2.2; *p* = 0.813) (Figure 5).

**Figure 6:**
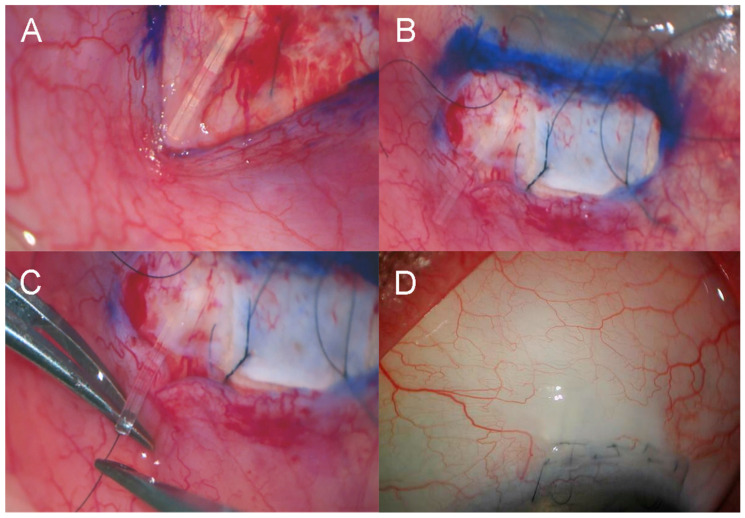
(**A**) Fornix-based conjunctival flap opened to demonstrate fibrosis occluding the proximal end of the PreserFlo. (**B**) Trabeculectomy flap with buried releasable 10/0 nylon sutures and exposed proximal end of PreserFlo. (**C**) 10/0 nylon intraluminal stent insertion to reduce risk of early post-operative hypotony and adjustment of outflow. (**D**) Post-operative appearance at 12 months with an IOP of 10 mmHg.

In PF eyes, 7 (14.6%) required surgical revision. One eye (2.1%) underwent Paul tube implantation, and the other six (12.5%) had open revision with trabeculectomy/MMC (Figure 6). Two (4.2%) occurred between 2 and 3 months, with the remaining five (10.4%) having between 11 and 13 months post-operatively. Late trabeculectomy complications included numerical hypotony in five eyes (10.6%), clinically significant hypotony in one eye (2.1%), and blebitis/endophthalmitis in one eye (2.1%). One eye (2.1%) underwent Baerveldt tube implantation with concurrent phacoemulsification and posterior chamber intraocular lens (PCIOL) insertion, and the other one (2.1%) required sequential CyPass implantation, phacoemulsification/PCIOL, and placement of a Baerveldt tube (Table 4).

## 4. Discussion

This retrospective cohort analysis compares the effectiveness and safety of PF and trabeculectomy for surgical glaucoma management. The findings indicate that both procedures reduce IOP and the need for medication.

Early post-operative hypotony, primarily occurring within the first two weeks, was common in PF eyes that did not receive an intraluminal 10/0 nylon suture. While most cases were asymptomatic and resolved spontaneously, some patients experienced complications like choroidal detachment and reduced visual acuity. To address this, the use of 10/0 nylon stenting was systematically adopted in the remaining 40 PF eyes, which effectively prevented early hypotony. Although approximately 75% of these stents were removed within three months, several cases maintained adequate IOP control with the stents in place. This suggests that without the stent, hypotony would have likely occurred. By the 12-month follow-up, no PF patients in this study had numerical hypotony. These results are consistent with the existing literature. Lupardi et al. reported no hypotony in stented PF eyes, while non-stented cases had a 28.6% incidence of hypotony and a 9.5% rate of choroidal detachment [6]. Similarly, Chami et al. found significantly higher rates of both numerical and symptomatic hypotony in non-stented PF eyes compared to their stented counterparts (44% vs. 24%, *p* = 0.007 and 28% vs. 13%, *p* = 0.027, respectively) [7]. Both studies advocate for the use of 10/0 nylon intraluminal stents to reduce hypotony. The use of 9/0 nylon, which provides total occlusion and must be removed to achieve drainage, has also been reported in the literature [7].

The concentration of MMC used in this PF series aligns with the 0.3–0.4 mg/mL concentration recommended in most of the literature [1,4,5,8]. Previous studies, such as one by Beckers et al., have demonstrated that a higher dose (0.4 mg/mL) results in better outcomes, including a higher rate of medication-free patients (90.3% vs. 51.9% at 0.2 mg/mL) and superior IOP reduction [9]. A recent study utilized sub-Tenon MMC administration over sponge-based application [10]. In the current cohort, the surgeon (G.A.L.) assessed the appropriate MMC dose at the time of surgery, tailoring it to the integrity of the conjunctival and Tenon’s tissue. While crucial for surgical success, overdosing MMC can lead to complications such as avascular conjunctiva and blebitis, particularly in elderly Caucasian patients [11,12].

The PF procedure in this study positioned the pledgets more posteriorly than in trabeculectomy, a technique that aligns with the resulting posterior position of PF blebs [11]. This may be a clinical advantage, as posterior bleb locations are associated with a lower risk of long-term complications like blebitis and endophthalmitis [11,12]. The findings indicated that fibrosis around the device tip in PF eyes may have limited the effectiveness of needling and potentially contributed to the requirement for open revision. In contrast, bleb needling for failed trabeculectomy can effectively puncture the Tenon’s cyst wall and fibrosis around the scleral flap, recreating the sclerotomy.

Trabeculectomy necessitates more frequent early post-operative visits (one to two weekly) to assess the need for 5-fluoruracil (5-FU) injections, massage of the scleral trapdoor, and potential suture removal. The less invasive nature of the PF procedure leads to less inflammation and fewer required clinical reviews, improving short-term patient quality of life [4]. At 12 months, however, trabeculectomy resulted in greater IOP reduction, which is the more clinically beneficial outcome, although it did not reach statistical significance in this study. This is likely due to the higher outflow through the trapdoor and the absence of a stent foreign body. Overall, PF success rates range from 52% to 74% at one year [5,8,9,13]. Baker et al. found that the probability of success was significantly higher in the trabeculectomy group (72.7%) compared to the PF group (53.9%, *p* < 0.01) [14]. Fili et al. reported that 94% of trabeculectomy eyes had a >20% reduction in IOP without medication, compared to 81% of PF eyes [1]. Similarly, Zweifel et al. found that trabeculectomy led to a greater IOP reduction (12 mmHg vs. 7 mmHg, *p* = 0.01) [15].

Postoperative inflammation is generally milder in PF patients, allowing for a more rapid steroid taper [4]. However, ceasing topical steroids earlier might be a contributing factor to the increased rates of fibrosis observed in PF eyes. Early hypotony in PF is often due to peritubular leaks, most of which resolve spontaneously within one to two weeks, especially if the flange is well-positioned. However, persistent hypotony can cause complications like anterior chamber flattening and choroidal detachment, requiring surgical revision. This study showed a higher rate of long-term hypotony in the trabeculectomy group. Although this, by definition, was regarded as a failure, only one eye required scleral patching due to reduced vision from choroidal folds [1,3,14,16,17].

PF eyes in this study showed a trend toward a higher rate (*p* = 0.059) of open revision. At revision surgery, fibrosis was commonly found surrounding the tip of the tube. This was addressed by either removing the PF or re-stenting it with a buried releasable 10/0 nylon suture, as well as performing a trabeculectomy with MMC adjacent to the original site (Figure 6). Other studies support open revision with MMC as a safe and effective alternative to device exchange or conversion to trabeculectomy [18,19]. Meta-analytic data have shown that PF patients, particularly those without prior glaucoma surgery, have increased re-intervention rates [5]. This persistent fibrosis remains a significant obstacle to surgical success and highlights the need for further research into anti-fibrotic interventions and myofibroblast inhibition [20].

When comparing trabeculectomy with the PF in a single-surgeon consecutive series, the surgeon’s consistent technique and patient selection offer a key advantage, minimizing the influence of inter-surgeon variability on outcomes. However, this study design is susceptible to significant biases. For example, the surgeon might initially perform PF on patients with less severe glaucoma or those with a higher chance of success, leading to a selection bias that could artificially inflate the new technique’s reported efficacy. Selection bias resulting from the PF cohort’s older age may explain their improved post-operative outcomes, which could be attributed to the reduced fibrotic response in this demographic. Furthermore, the predominantly Caucasian patient population in both cohorts limits the study findings and may have contributed to increased rates of success. A temporal bias is also present; any improvements in surgical instruments, technique or post-operative management protocols over time would benefit the later PF cohort, confounding the direct comparison with the earlier trabeculectomy group. As a result, the findings of this study may not be generalizable to other surgeons or provide a truly objective comparison of the two techniques that would be better achieved with a multi-center, multi-surgeon, randomized trial with larger sample sizes.

## 5. Conclusions

In conclusion, this study confirms that both PF and trabeculectomy are effective surgical options for glaucoma, significantly reducing IOP and medication dependency. The PF procedure was associated with less inflammation and a more rapid steroid taper. The adoption of intraoperative nylon stenting in PF surgery proved crucial in preventing early post-operative hypotony. However, PF eyes had a higher rate of open surgical revisions, often due to persistent fibrosis that limited the effectiveness of needling. Future research should focus on refining patient selection criteria for PF and exploring novel anti-fibrotic interventions to improve the long-term success rates.

## Figures and Tables

**Table 1:** Patient characteristics.

	PF (*n* = 48)	Trabeculectomy (*n* = 47)	*p* Value
Eye—right (%)	24 (50.0)	27 (57.5)	0.467
Sex—male (%)	25 (52.1)	21 (44.7)	0.470
Age, mean (years) (SD)	72.9 (9.6)	65.3 (13.8)	0.002
Ethnicity			0.211
Caucasian (%)	45 (93.8)	41 (87.2)	
Asian (%)	2 (4.2)	6 (12.8)	
Other (%)	1 (2.1)	0 (0.0)	
Glaucoma severity			0.034
Mild (%)	16 (34.8)	27 (57.4)	
Moderate (%)	9 (19.6)	10 (21.3)	
Severe (%)	21 (45.7)	10 (21.3)	
Glaucoma type			0.439
POAG (%)	35 (72.9)	39 (83.0)	
PACG (%)	3 (6.3)	1 (2.1)	
Other (%)	10 (20.8)	7 (14.9)	
Pre-operative IOP			0.174
≤21 mmHg (%)	24 (50.0)	17 (36.2)	
>21 mmHg (%)	24 (50.0)	30 (63.8)	
Pre-operative IOP, mean (mmHg) (SD)	23.0 (6.2)	26.5 (8.1)	0.020
Pre-operative medications			0.355
≤3 (%)	22 (45.8)	26 (55.3)	
4 or 5 (%)	26 (54.2)	21 (44.7)	
Number of medications, mean (SD)	3.5 (1.2)	3.4 (0.9)	0.473
Diamox (%)	8 (16.7)	16 (34.0)	0.051
Best corrected visual acuity, mean (SD)	−0.75 (0.26)	−0.76 (0.29)	0.861
Mean deviation(dB) (SD)	−11.1 (7.8)	−7.3 (6.6)	0.014
Lens—phakic (%)	26 (54.2)	22 (46.8)	0.473
Surgical ocular history	35 (76.1)	37 (80.4)	0.613
	SLT 26	SLT 20	
	YAG Laser 13	YAG Laser 12	
	Vitrectomy 2	Vitrectomy 1	
Operation details			
Intraluminal stenting			
Stenting (%)	40 (83.3)	-	
Removal (%)	31 (77.5)	-	
Time to removal, mean (weeks) (SD)	7.8 (10.5)	-	
Time to removal, median (weeks) (interquartile range)	4 (2, 8)	-	
MMC (%)			0.131
0.01 (%)	2 (4.2)	1 (2.1)	
0.02 (%)	17 (60.4)	20 (42.6)	
0.03 (%)	29 (60.4)	20 (42.6)	

Statistical analysis: Proportions, Chi-squared test or Fisher’s exact test; means, independent t-test. PF: Preserflo Microshunt, POAG: primary open-angle glaucoma, PACG: primary angle closure glaucoma, IOP: intraocular pressure, MMC: mitomycin C, SLT: Selective Laser Trabeculoplasty, YAG: Yttrium–Aluminum–Garnet.

**Table 2:** Estimated mean IOP and changes in mean IOP using inverse probability weighted adjustment.

Time Point	PF Mean IOP	Δ Within PF *	Trab Mean IOP	Δ Within Trab *	Δ Between Groups	*p* Value (Between Groups)	95% CI
Preop	24.9		25				
Day 1	8.6	−16.3	15.6	−9.5	−6.8	0.004	−11.5, −2.2
Week 1	9.7	−15.2	13.4	−11.6	−3.6	0.086	−7.7, 0.5
Month 1	12.7	−12.2	10.4	−14.6	2.4	0.247	−1.7, 6.5
Month 3	11.4	−13.5	9.9	−15.1	1.6	0.468	−2.7, 5.9
Month 6	12.1	−12.8	9.2	−15.9	3	0.145	−1.1, 7.2
Month 12	13.1	−11.8	10.3	−14.7	2.9	0.246	−2.0, 7.9

Multilevel mixed-effects linear regression controlled for age, mean deviation, pre-operative IOP, and number of glaucoma medications. * *p* < 0.05 within group.

**Table 3:** Estimated mean number of medications and changes in mean number of medications using inverse probability weighted adjustment.

Time Point	PF Mean Meds	Δ Within PF *	Trab Mean Meds	Δ Within Trab *	Δ Between Groups	*p* Value (Between Groups)	95% CI
Preop	3.43		3.50				
Week 1	0.21	−3.21	0.09	−3.41	0.20	0.284	−0.16, 0.56
Month 1	0.07	−3.36	0.17	−3.33	−0.03	0.848	−0.38, 0.32
Month 6	0.21	−3.22	0.18	−3.32	0.10	0.629	−0.31, 0.51
Month 12	0.54	−2.88	0.20	−3.30	0.41	0.138	−0.13, 0.96

Multilevel mixed-effects Poisson regression controlled for age, pre-operative IOP, number of glaucoma medications, and mean deviation. * *p* < 0.05 within group.

**Table 4:** Complications during the 12-month follow-up period.

Complication	PF (*n* = 48)	Trab (*n* = 47)	*p*-Value
Early numerical hypotony (%)	6 (14.6)	6 (12.8)	1.00
Late IOP ≤ 5 mmHg (%)	0 (0)	6 (12.8) (5 numerical hypotony)	0.012
Clinically significant hypotony with choroidal effusion (%)	1 (2.1)	1 (2.1)	1.00
Macroscopic hyphaema (%)	2 (4.2)	1 (2.1)	1.00
Intracameral tube truncation (%)	1 (2.1)	N/A	
Extrusion (%)	1 (2.1)	N/A	
Bleb Leak (%)	0 (0%)	2 (4.2)	0.242
Blebitis/endophthalmitis (%)	0 (0%)	1 (2.1)	0.495
Peripheral iris incarceration (%)	0 (0%)	1 (2.1)	0.495
Bleb needling			
Eyes that had needling (%)	16 (33.3)	13 (27.7)	0.548
No. of needlings in the group, mean (SD)	0.6 (0.9)	0.4 (0.7)	0.344
No. of needlings in eyes that had needlings, mean (SD)	1.6 (0.7)	1.5 (0.7)	0.622
No. of needlings in eyes that had needlings, median (IQR)	1 (1, 2)	1 (1, 2)	
Time (months) to first needling, mean (SD)	2.6 (2.9)	1.9 (3.2)	0.531
Time (months) to first needling, median (IQR)	1.2 (0.4, 3.7)	0.9 (0.5, 1.3)	
Additional glaucoma surgeries (%)	10 (20.8)	2 (4.3)	0.027
Open Revision (%)	7 (14.6)	2 (4.2)	0.059

## Data Availability

Data can be made available from the authors if given reasonable written request.

## Abbreviations

The following abbreviations are used in this manuscript:

PF	PreserFlo MicroShunt
IOP	Intraocular Pressure
5-FU	5-Fluorouracil
MMC	Mitomycin C
VA	Visual Acuity
IPTW	Inverse probability of treatment weighting

## References

[1] Fili S., Boutsika K., Kokkali S., Patsoukis A., Patsouras A., Laspas P., Garnavou-Xirou C., Georgopoulos G. (2022). PreserFlo™ MicroShunt Versus trabeculectomy in Patients with Moderate to Advanced Open-Angle Glaucoma: 12-Month Follow-Up of a Single-Center Prospective Study. Cureus.

[2] Jamke M., Fritz M., Laspas P., Pfeiffer N., Grus F.H., Hoffmann E.M. (2023). PRESERFLO™ MicroShunt versus trabeculectomy: 1-year results on efficacy and safety. Graefes Arch. Clin. Exp. Ophthalmol..

[3] Pillunat K.R., Herber R., Jasper C., Berchner J., Pfeiffer N., Pillunat L.E. (2022). PRESERFLO™ MicroShunt versus trabeculectomy: First results on efficacy and safety. Acta Ophthalmol..

[4] Khan A., Khan A.U. (2024). Comparing the safety and efficacy of PreserFlo Microshunt implantation and trabeculectomy for glaucoma: A systematic review and meta-analysis. Acta Ophthalmol..

[5] Governatori L., Oliverio L., Mermoud A., Scampoli A., Sarati F., Carradori A., Catalani R., Monaco C., Caporossi T., Rizzo S. (2025). PreserFlo MicroShunt versus trabeculectomy: An updated meta-analysis and systematic review. Graefes Arch. Clin. Exp. Ophthalmol..

[6] Lupardi E., Laffi G.L., Moramarco A., Barboni P., Fontana L. (2023). Systematic PreserFlo MicroShunt Intraluminal Stenting for Hypotony Prevention in Highly Myopic Patients: A Comparative Study. J. Clin. Med..

[7] Chami J., Tan J.C.K., Manning D., Weiss J., Schlenker M.B., Ahmed I.I.K. (2025). Comparative Study of Early Safety and Effectiveness Outcomes of the PreserFlo MicroShunt with and without an Intraluminal Suture Stent. Ophthalmol. Glaucoma.

[8] Bhayani R., Jawla S., Trikha S., Mehta R., Ahir B.K., Lyall D.A.M., Chandra A., Ahmed F., Smith H.B. (2023). Short-term safety and efficacy of PreserFlo™ Microshunt in glaucoma patients: A multicentre retrospective cohort study. Eye.

[9] Beckers H.J.M., Aptel F., Webers C.A.B., Bluwol E., Martinez-de-la-Casa J.M., Cordeiro M.F., Stalmans I., Vandewalle E., García-Feijoó J. (2022). Safety and Effectiveness of the PRESERFLO^®^ MicroShunt in Primary Open-Angle Glaucoma: Results from a 2-Year Multicenter Study. Ophthalmol. Glaucoma.

[10] Majtanova N., Takacova A., Kurilova V., Hejsek L., Majtan J., Kolar P. (2025). One-Year Comparison of Efficacy and Safety of PreserFlo MicroShunt with Mitomycin C Applied by Sub-Tenon Injection Versus Sponge. Ophthalmol. Ther..

[11] Ibarz Barberá M., López-Grau N.S., Amaro-Alcázar A., Morales-Fernandez L., García-Feijoó J., Martínez-de-la-Casa J.M. (2023). Bleb geometry and morphology after PreserFlo Microshunt surgery: Risk factors for surgical failure. PLoS ONE.

[12] Hasan S.M., Theilig T., Meller D. (2023). Comparison of Bleb Morphology following PRESERFLO^®^ MicroShunt and trabeculectomy Using Anterior Segment OCT. Diagnostics.

[13] Tanner A., Haddow J., Ahir B.K., Lyall D.A.M., Jawla S., Ahmed F., Chandra A., Bhayani R. (2023). One-year surgical outcomes of the PreserFlo MicroShunt in glaucoma: A multicentre analysis. Br. J. Ophthalmol..

[14] Baker N.D., Barnebey H.S., Moster M.R., Flowers B.E., Maslin J.S., Grover D.S., Panarelli J.F., Sarkisian S.R., Noecker R.J., Kahook M.Y. (2021). Ab-Externo MicroShunt versus Trabeculectomy in Primary Open-Angle Glaucoma: One-Year Results from a 2-Year Randomized, Multicenter Study. Ophthalmology.

[15] Zweifel L.A.B., Storp J.J., Vietmeier F.E., Eter N., Brücher V.C. (2024). PreserFlo MicroShunt versus Trabeculectomy: Efficacy and Surgical Success within a Heterogenous Patient Cohort. Life.

[16] Nobl M., Grün C., Kassumeh  S., Priglinger S., Mackert M.J. (2023). One-Year Outcomes of PreserFlo™ MicroShunt Implantation versus trabeculectomy for Pseudoexfoliation Glaucoma. J. Clin. Med..

[17] Batlle J.F., Fantes F., Riss I., Pinchuk L., Alburquerque R., Kato Y.P., Arrieta E., Peralta A.C., Palmberg P., Parrish R.K. (2021). Long-term Results of the PRESERFLO MicroShunt in Patients with Primary Open-angle Glaucoma from a Single-center Nonrandomized Study. J. Glaucoma.

[18] Sadruddin O., Pinchuk L., Angeles R., Palmberg P., Parrish R.K., Weber B.A., Mehra K.S. (2019). Ab externo implantation of the MicroShunt, a poly (styrene-block-isobutylene-block-styrene) surgical device for the treatment of primary open-angle glaucoma: A review. Eye Vis..

[19] Strzalkowska A., Al-Mugheiry T.S., Trikha S., Verma S., Stalmans I., Dick A.D. (2023). Outcomes of Open Bleb Revision After PreserFlo MicroShunt Failure in Patients with Glaucoma. J. Glaucoma.

[20] Saeed E., Gołaszewska K., Dmuchowska D.A., Zalewska R., Konopińska J. (2023). The PreserFlo MicroShunt in the Context of Minimally Invasive Glaucoma Surgery: A Narrative Review. Int. J. Environ. Res. Public Health.

